# GliMR: Cross-Border Collaborations to Promote Advanced MRI Biomarkers for Glioma

**DOI:** 10.1007/s40846-020-00582-z

**Published:** 2020-12-03

**Authors:** Patricia Clement, Thomas Booth, Fran Borovečki, Kyrre E. Emblem, Patrícia Figueiredo, Lydiane Hirschler, Radim Jančálek, Vera C. Keil, Camille Maumet, Yelda Özsunar, Cyril Pernet, Jan Petr, Joana Pinto, Marion Smits, Esther A. H. Warnert

**Affiliations:** 1 Ghent Institute for Metabolic and Functional Imaging (GIfMI), Ghent University, Ghent, Belgium; 2 School of Biomedical Engineering & Imaging Sciences, King’s College London, St Thomas’ Hospital, London, SE1 7EH UK; 3 Department of Neuroradiology, King’s College Hospital NHS Foundation Trust, London, SE5 9RS UK; 4 Department of Neurology, University Hospital Centre Zagreb, Zagreb, Croatia; 5 Division of Radiology and Nuclear Medicine, Department of Diagnostic Physics, Oslo University Hospital, Oslo, Norway; 6 Institute for Systems and Robotics - Lisboa and Department of Bioengineering, Instituto Superior Técnico, Universidade de Lisboa, Lisbon, Portugal; 7 Department of Radiology, C.J. Gorter Center for High Field MRI, Leiden University Medical Center, Leiden, The Netherlands; 8 Department of Neurosurgery, St. Anne’s University Hospital and Medical Faculty, Masaryk University, Brno, Czech Republic; 9 Department of Radiology, Amsterdam University Medical Center, VUmc, Vrije Universiteit Amsterdam, Amsterdam, The Netherlands; 10 Inria, Univ Rennes, CNRS, Inserm, Rennes, France; 11 Department of Radiology, Faculty of Medicine, Adnan Menderes University, Aydın, Turkey; 12 Centre for Clinical Brain Sciences & Edinburgh Imaging, University of Edinburgh, Edinburgh, UK; 13 Institute of Radiopharmaceutical Cancer Research, Helmholtz-Zentrum Dresden-Rossendorf, Dresden, Germany; 14 Department of Engineering Science, Institute of Biomedical Engineering, University of Oxford, Oxford, UK; 15 Department of Radiology & Nuclear Medicine, Erasmus MC, Rotterdam, The Netherlands

**Keywords:** Glioma, Advanced MRI, Multi-disciplinary, Networking, Translational research, COST action

## Abstract

**Purpose:**

There is an annual incidence of 50,000 glioma cases in Europe. The optimal treatment strategy is highly personalised, depending on tumour type, grade, spatial localization, and the degree of tissue infiltration. In research settings, advanced magnetic resonance imaging (MRI) has shown great promise as a tool to inform personalised treatment decisions. However, the use of advanced MRI in clinical practice remains scarce due to the downstream effects of siloed glioma imaging research with limited representation of MRI specialists in established consortia; and the associated lack of available tools and expertise in clinical settings. These shortcomings delay the translation of scientific breakthroughs into novel treatment strategy. As a response we have developed the network “Glioma MR Imaging 2.0” (GliMR) which we present in this article.

**Methods:**

GliMR aims to build a pan-European and multidisciplinary network of experts and accelerate the use of advanced MRI in glioma beyond the current “state-of-the-art” in glioma imaging. The Action Glioma MR Imaging 2.0 (GliMR) was granted funding by the European Cooperation in Science and Technology (COST) in June 2019.

**Results:**

GliMR’s first grant period ran from September 2019 to April 2020, during which several meetings were held and projects were initiated, such as reviewing the current knowledge on advanced MRI; developing a General Data Protection Regulation (GDPR) compliant consent form; and setting up the website.

**Conclusion:**

The Action overcomes the pre-existing limitations of glioma research and is funded until September 2023. New members will be accepted during its entire duration.

## State-of-the-Art MR Imaging for Glioma

In Europe, approximately 50,000 new cases of glioma (brain tumours originating from glial cells) occur each year [1], with numbers constantly rising with an aging European population. Median survival ranges from more than 10 years for low-grade glioma, to only 14.6 months for the most aggressive forms of glioma, namely glioblastoma [2]. Survival increases when the tumour is diagnosed accurately early on and an appropriate course of treatment is applied. However, screening and management are limited due to the heterogeneity in tumour growth dynamics, as well as the high inter- and intra-tumoral biological spatial heterogeneity [3]. Currently, there is increasing interest in advanced imaging techniques to identify the most malignant regions within the tumour according to the 2016 World Health Organisation (WHO) Brain Tumour Classification [4]. This understanding will help shape the highly-personalised therapy necessary for glioma and allow for the development of biomarkers for early tumour diagnosis and treatment planning [3].

**Fig. 1:**
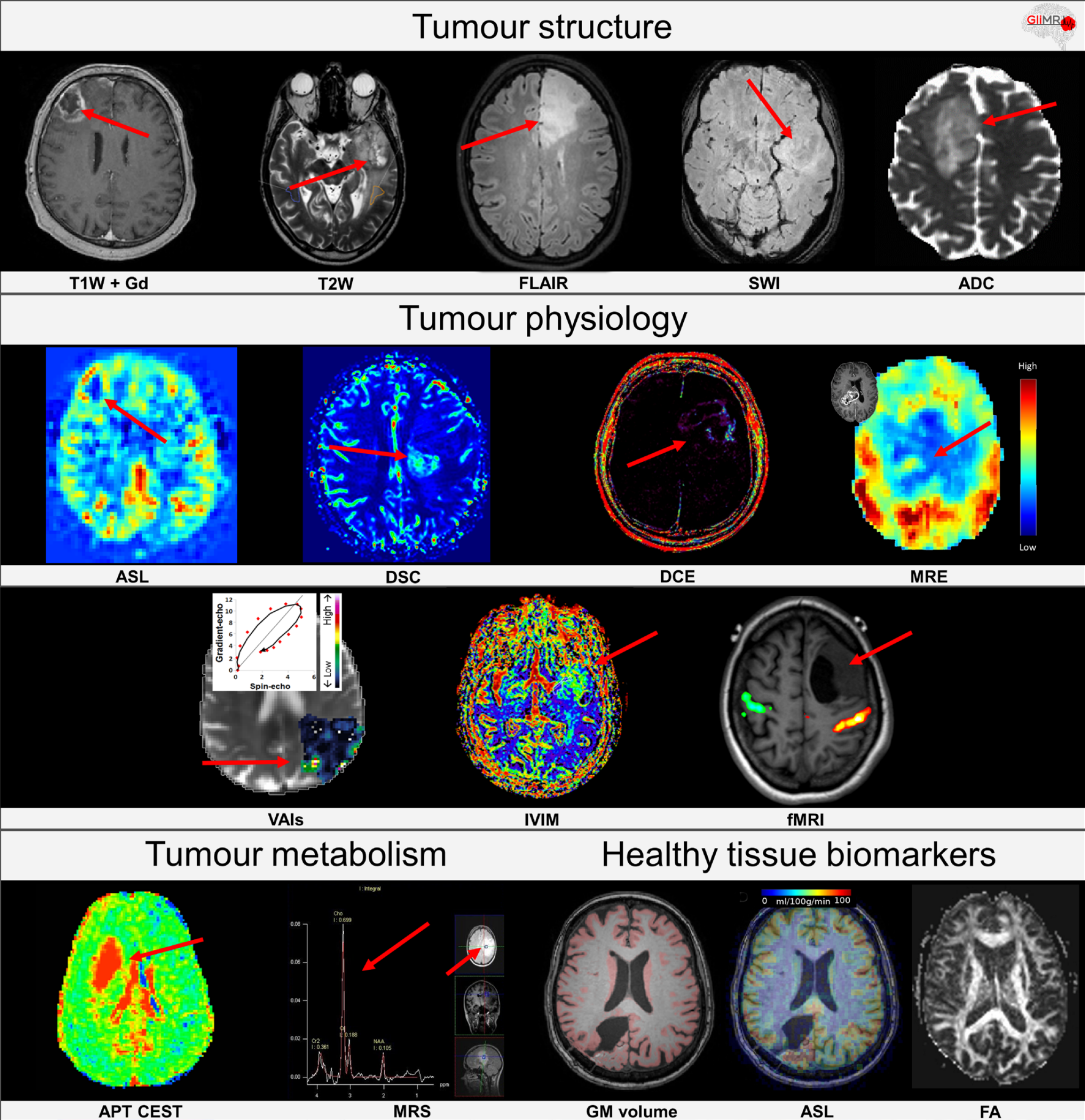
Examples of conventional and advanced MRI methods. Biomarkers highlighting tumour structure from left to right: T1-weighted after injection of a gadolinium based contrast agent, T2-weighted, fluid-attenuated inversion recovery (FLAIR), susceptibility weighted imaging (SWI), apparent diffusion coefficient (ADC); biomarkers highlighting tumour physiology: arterial spin labeling (ASL), dynamic susceptibility contrast (DSC), dynamic contrast enhanced (DCE), magnetic resonance elastography (MRE), vessel architectural imaging (VAI), intravoxel incoherent motion (IVIM) perfusion, functional MRI (fMRI); biomarkers highlighting tumour metabolism: amide proton transfer chemical exchange saturation technique (APT CEST), magnetic resonance spectroscopy; biomarkers highlighting healthy tissue biomarkers: grey matter (GM) volume, ASL, and fractional anisotropy (FA) [34, 70]

Magnetic resonance imaging (MRI) is the diagnostic modality of choice to investigate the structure and physiology of brain tissue. It is a widely available and non-invasive medical imaging tool and the only modality to deliver information of brain tissue at a sufficient contrast, spatial and temporal resolution [5, 6]. While conventional MRI techniques assess anatomical information about the structure of brain tissue and vasculature, advanced MRI techniques can measure dynamic and functional processes such as perfusion [using arterial spin labeling (ASL), dynamic contrast enhanced (DCE), and dynamic susceptibility contrast (DSC)] [7, 8], metabolism [using MR spectroscopy (MRS)] [9], microstructure [using diffusion kurtosis imaging (DKI), intravoxel incoherent motion (IVIM) [10], and other diffusion MRI techniques] [11–13], stiffness [using magnetic resonance elastography (MRE)] [14], oxygen extraction [using BOLD-MRI] [15], and vessel architecture [using vessel architectural imaging (VAI)] [16]. Examples of these conventional and advanced MRI techniques are provided in Fig. 1. The resulting quantitative information can be used to categorize and discriminate gliomas better than using anatomical image sequences alone [6].

The development of advanced techniques to become MRI biomarkers has progressed through the combination of MRI approaches (multi-modality) and through data-driven approaches to combine images with genetic and clinical information—where “radiomics” often plays a vital role [9, 17, 18]. Growing evidence highlights the importance of combining biomarkers to non-invasively map the heterogeneous microenvironment of brain tumours [19–21]. This is critical for a more reliable glioma assessment and, therefore, an optimally individualized treatment approach [19, 20]. Additionally, the extraction of a large number of informative features from imaging, genetics, and clinical assessments that are subjected to machine learning-based analyses (radiomics), can be used to aid diagnosis and to predict treatment response for an individual patient [20, 22]. This approach is highlighted by the WHO classification of gliomas updated in 2016 [4] which stated that the key prognostic determinants in glioma are genomic and proteomic, rather than histopathological, analyses including mutations in isocitrate dehydrogenase (IDH), alpha-thalassemia/mental retardation syndrome X-linked (ATRX), tumour protein p53 and O^6^‐alkylguanine DNA‐alkyltransferase MGMT genes and 1p/19q co-deletion which have already been shown to influence glioma malignancy and treatment response [4, 23].

In clinical practice, some advanced MRI techniques are already frequently used to support the monitoring of patients diagnosed with glioma before, during, and after treatment [8]. MRI offers the ability to perform non-invasive, repeated examinations with minimum risk for the patient [6, 8]. First, advanced MRI is optimal for target delineation during radiotherapy (RT) treatment planning, as novel MRI biomarkers lead to the improved localisation and determination of the extent of the tumour [24]. For example, diffusion kurtosis imaging (DKI) may detect early anomalies in tissue microstructure by tumour infiltration, not depicted on morphological MRI [11, 13]. Moreover, MRI can also be used for RT treatment planning instead of computed tomography (CT) which reduces radiation exposure and avoids MRI to CT co-registration errors. While the attenuation property of tissue is directly linked to the voxel intensity in CT, with MRI it is indirectly linked and typically has to be estimated by a combination of atlas-based and intensity-based machine learning methods, usually using T1-weighted or Ultra-short Echo-Time (UTE) sequences [25]. Secondly, treatment monitoring is important for patients diagnosed with low-grade glioma, for whom early and aggressive treatments do not necessarily lead to improved overall survival [26]. Novel physiological parameters resulting from advanced MRI techniques, such as ^1^H-MRS and DSC, can serve as early markers for progression from low to high-grade glioma [27]. In high grade glioma, the accurate determination of treatment response to distinguish tumour progression from radiation necrosis and pseudoprogression is vital for management decisions [8, 28]. In contrast to conventional MRI, advanced MRI techniques, such as chemical exchange saturation transfer (CEST), MRS and MR perfusion, have shown the potential to distinguish treatment effects, such as radiation necrosis and pseudoprogression, from true tumour progression. Thus, the application of those techniques greatly improves timely and accurate decisions on further patient management [29–31]. Lastly, glioma treatment is associated with brain damage, both within and remote from the primary target region of treatment [29, 32]. Studies are emerging that investigate the side-effects of different treatment strategies (e.g. radiation, chemotherapy, or both) on the brain structure and function, which lead to a reduced quality of life.

For research purposes, a plethora of different advanced MRI methods has been explored to help investigate, develop, and improve novel treatment strategies for glioma. More specifically, advanced MRI techniques are being used to study in vivo glioma pathophysiology, for example, using CEST [33]. Additionally, the techniques have the potential to identify novel targets for treatment [28] and are used to investigate mechanisms of treatment effects both on pathological and healthy tissue using MR techniques such as ASL, DCE, and diffusion MRI [32, 34, 35]. As an example, non-invasive physiological biomarkers are being developed for the early identification of adverse treatment effects on healthy tissues [34]. Whilst current practice mitigates the effects of RT by avoiding where possible exposure of the brainstem, hippocampus, and cranial nerves [36], little is known about individual tolerance to chemo-radiation and its association with long-term changes in cognition and quality of life [32, 37]. Biomarkers, based on advanced MRI, have the potential to better identify structures at risk and detect adverse effects at an early stage. This approach could allow personalised therapy and facilitate a timely therapy adaptation to minimize adverse effects. Furthermore, advanced MRI has great potential for use in clinical trials by optimising patient selection by exploiting the excellent sensitivity of MRI to the heterogeneous microenvironment of glioma, as well as the capability for treatment response monitoring and differentiating treatment response from tumour progression [8, 28].

In summary, advanced MRI techniques might aid in the detection, diagnosis, treatment planning, assessment of treatment response, and prognosis of gliomas and fulfill a key role in the personalised management of glioma in clinical practice, as well as in many research initiatives focussing on the mechanisms of pathophysiology and novel treatment strategies.

## Lack of Harmonization Hampers Widespread Use in Research and Clinic

Despite its clear potential, and with the exception of diffusion-weighted imaging (DWI) [38] and dynamic susceptibility contrast (DSC) [38, 39], advanced MRI methods are scarcely used in clinical practice of glioma diagnostics. The application of advanced MRI is hampered by a scattered research landscape for glioma-related MRI development, lack of tools readily available for clinical applications, and limited presence in established consortia for research and clinical application in glioma.

Regardless of the relatively high incidence of glioma in Europe [1], advanced MRI studies are often designed to answer specific questions, related to a single experimental sequence only. As a consequence, the acquired data may not be directly transferable to other institutions for use in larger multi-centre, multi-source studies aimed at validating these methods [40]. This is further aggravated by several factors causing even more fragmentation of research group efforts. Despite the efforts of some highly successful European research organisations, such as the European Organisation for Research and Treatment of Cancer (EORTC) [41], the research harmonisation at the pan-European level, equivalent to the National Institutes of Health (NIH) in the United States, is lacking. The lack of coordination between the efforts of research groups results in a scattered research landscape with a wide variety of local expertise and research focus [8, 38]. The current economic gradients contributing to the general “brain drain” towards northern and western Europe, concentrating the expertise in the bigger academic centres exacerbates this variety [42]. Local differences in languages and legislations further complicate inter-institutional exchange [43]. Although not immediately evident, this scattered research landscape might constrain patient participation in leading-edge research which limits recruitment numbers, causes selection bias and deprives patients from being able to contribute to scientific advancement.

Because advanced MRI methods are often resource-heavy and require unique expertise, MRI acquisition protocols may differ between sites or be excluded altogether in a research setting [8, 38]. Further inconsistencies in imaging are caused by the broad range of approaches for subsequent image analysis and interpretation [8, 38]. This is especially problematic if optimal in vivo characterisation of glioma should include multiple conventional and advanced MRI parameters [44].

The Brain Imaging Data Structure (BIDS) [45], Quantitative Imaging Network (QIN) [46], Quantitative Imaging Biomarkers Alliance (QIBA) [47], European Imaging Biomarker Alliance (EIBALL) [48] and Open Source Initiative for Perfusion Imaging (OSIPI) [49] initiatives have recently made efforts to develop standards for data acquisition, sharing, and processing. These endeavours comprise not only formulating recommendations for conventional MRI [50], but also for advanced MRI techniques in certain targeted patient populations. More specifically, this includes consensus recommendations for DSC in high-grade gliomas [51], brain perfusion imaging acquisition and processing recommendations for ASL [52, 53], or functional (f)MRI recommendations for pre-treatment planning and follow-up [54]. Despite such efforts, approaches for the harmonisation of data acquisition, recommendations for the optimal combination of MRI-biomarkers, and standardised open-source data analysis software for post-processing are currently not widely accepted, implemented or available.

Additionally, there are well-established consortia with a focus on glioma research, specifically focussing on new and improved treatment strategies. However, despite the presence of such collaborative networks throughout Europe or with European participation, such as EORTC [41], Response Assessment in Neuro-Oncology (RANO) [55], European Association of Neuro-Oncology (EANO) [56], Consortium to Inform Molecular and Practical Approaches to CNS Tumor Taxonomy (cIMPACT) [57], or Glioma Longitudinal Analysis Consortium (GLASS) [58] brain tumour groups, the European advanced MR imaging research community only has limited representation in these consortia. In conclusion, there is limited use of advanced MRI for glioma diagnostics because there is not an optimal critical mass of multidisciplinary experts in the field of glioma research and advanced MRI.

In summary, the current research conditions hamper advanced MRI to reach its full potential for glioma diagnostics. Ultimately, these shortcomings complicate the formation of big data sets, delay scientific breakthroughs for novel treatment strategy developments, complicate the development of radiomics approaches for glioma, and result in stagnating progress towards personalised medicine for this patient group. Large, longitudinal studies are key to finding associations between glioma treatment and MRI biomarkers as well as patient outcomes including long-term quality of life and cognitive capacity. These findings might lead to successful tools for patient monitoring and early prediction of patient outcomes.

## CA18206: Glioma MR Imaging 2.0

**Fig. 2:**
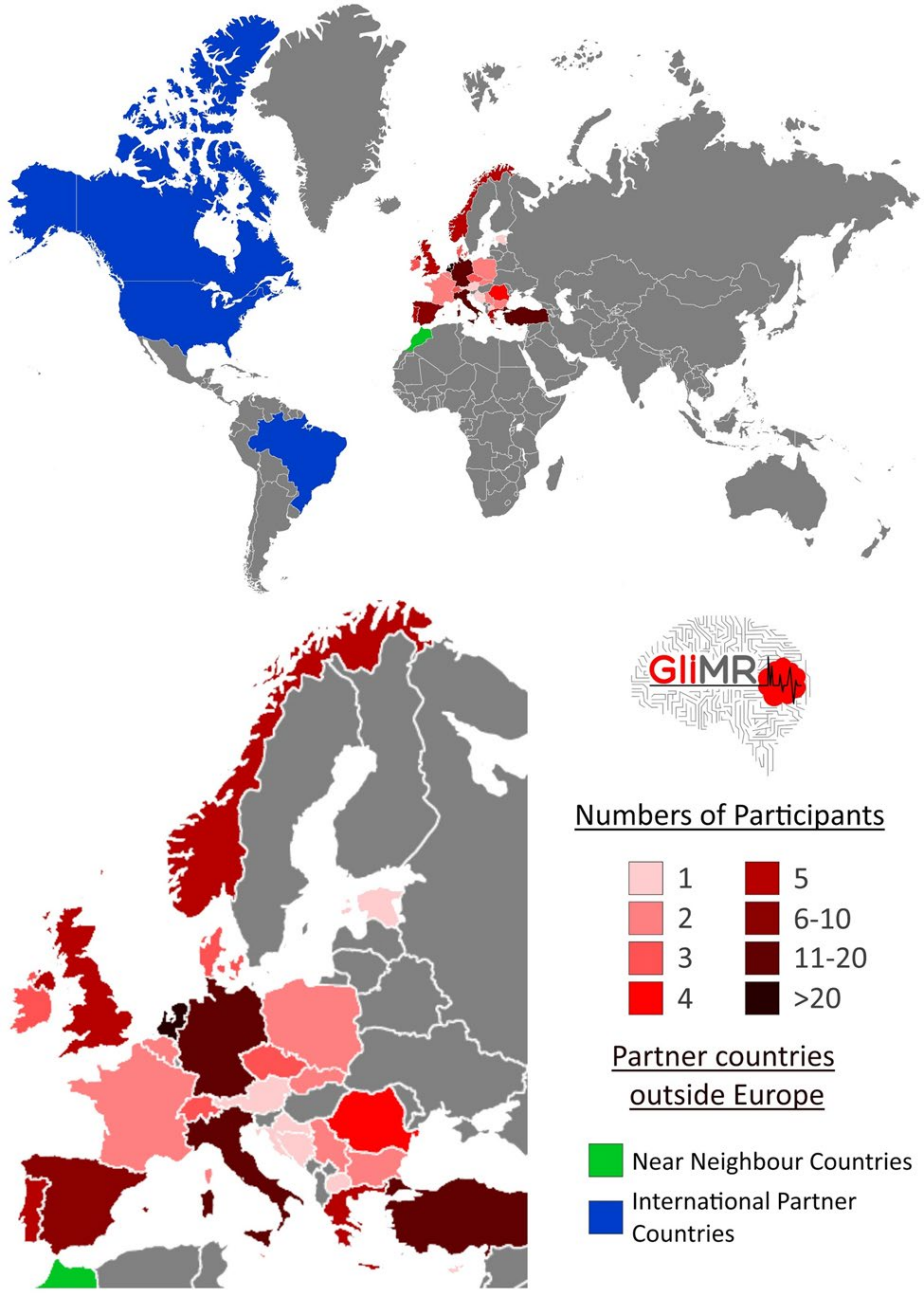
Geographical visualisation of GliMR’s participants within and outside Europe. Number of participants per European country is given, as well as the Near Neighbour Countries and the International Partner Countries [60]

To overcome these complex obstacles that hinder the progress of advanced MRI research in glioma, a group of European researchers strove to connect the imaging and non-imaging communities and to develop a powerful pan-European, multidisciplinary consortium of researchers and clinicians, patient organisations, data scientists, and MR imaging scientists. The proposed Action towards this goal, named ‘Glioma MR Imaging 2.0 (GliMR)’, was funded for four years (Sep 2019—Sep 2023) by the European Cooperation in Science and Technology (COST) in June 2019, at present connecting over 150 clinicians, engineers, and physicists from 26 European countries,^1^ one COST Near Neighbour Country (Morocco) and three International Partner Countries (Canada, the United States and Brasil), (Fig. 2) [59, 60].

**Fig. 3:**
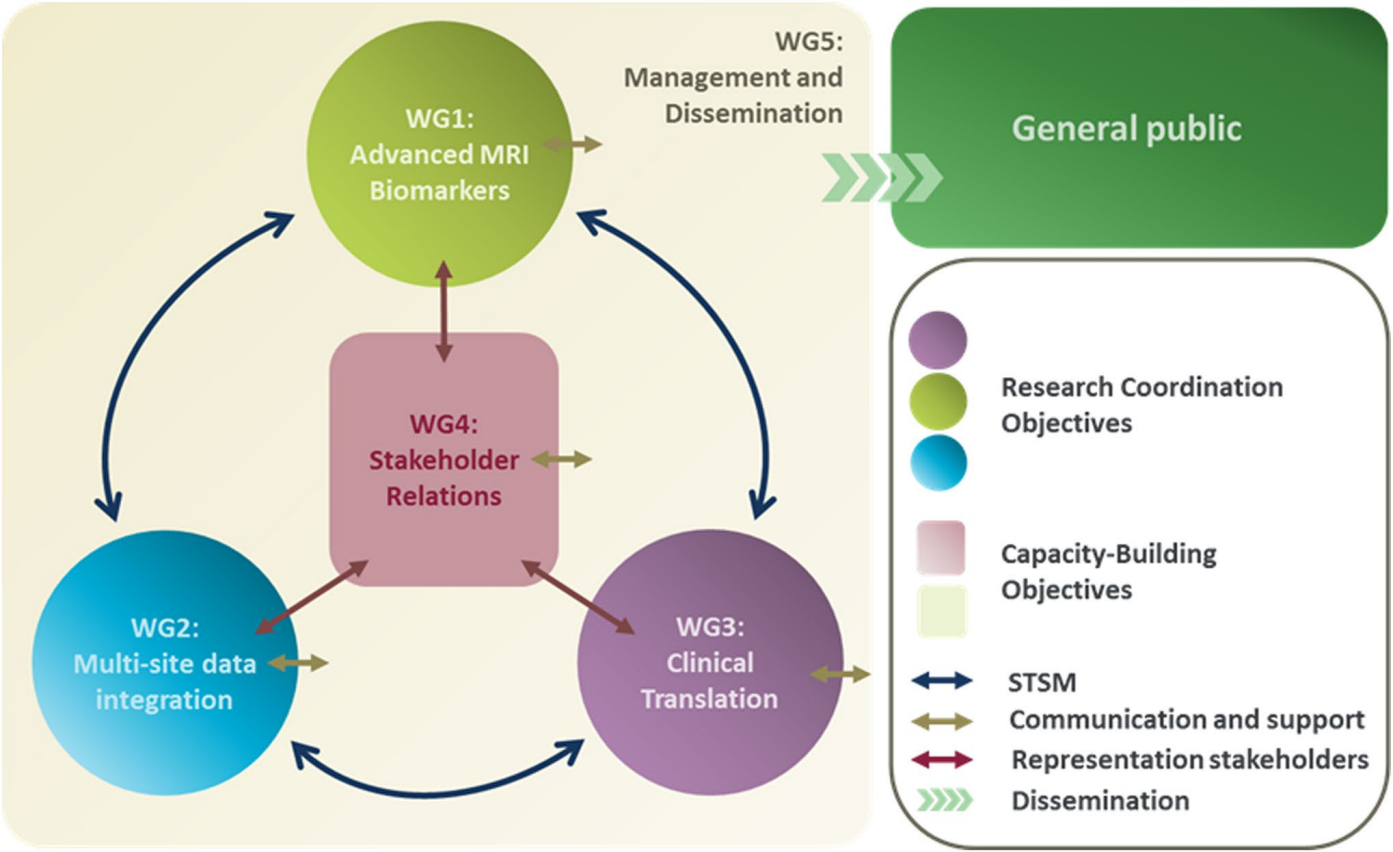
PERT chart of the structure within GliMR. Note the interaction between the Working Groups via various routes, denoted by coloured arrows. Communication between the Action and the general public will be ensured through Working Group 5

**Fig. 4:**
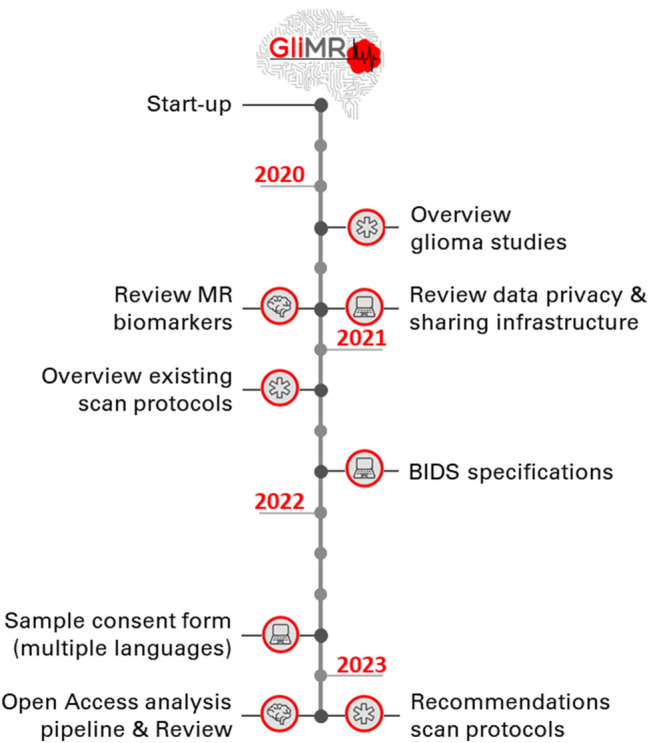
GliMR’s milestones throughout the 4-year lifespan of the Action, from June 2019 until April 2023. Each icon represents one of the Working Groups: ‘brain’: Advanced MRI biomarkers for glioma characterisation; ‘laptop’: ‘Multi-site data integration’; ‘star’: ‘Clinical translation’. The goals of the remaining two Working Groups are ongoing, therefore, not included in this timeline

GliMR focuses on creating a network in which the glioma imaging community within Europe is connected, coordinates the development of advanced MRI biomarkers and the collection of datasets across Europe, as well as stimulating their use in research and clinical settings. Through the organisation of international meetings and funding calls for research exchanges and conference attendance, the Action aims to reach a state of maximal progress in the development and application of advanced MR imaging for improved decision making in the diagnosis, patient monitoring, and assessment of treatment response in clinical trials and clinical practice. The Action aims to go beyond the state-of-the-art in glioma imaging by accelerating the use of advanced MRI for glioma in four focus areas: tumour characterisation, identification of regions at risk for progression, assessment of disease progression, and evaluation of treatment-related adverse effects. GliMR will apply new insights to stimulate innovation in personalised clinical management strategies, aiming at the refinement of diagnosis and the assessment of disease progression, the minimisation of adverse effects of treatment, and eventually the improvement of the long-term health-related quality of life of the patient. GliMR is structured in five Working Groups (Fig. 3), each with its own specific focus, aims and milestones, as summarized on the timeline in Fig. 4. All are collaborating to achieve the main goal of the Action: (1) Advanced MRI biomarkers for glioma characterisation, (2) Multi-site data integration, (3) Clinical translation, (4) Stakeholder relations, and (5) Dissemination.

The Working Group ‘Advanced MRI biomarkers for glioma characterisation’ focusses on the identification and quantification of advanced MRI biomarkers for application in glioma by sharing knowledge, as well as promoting best practices. Current knowledge on the most pertinent, existing, advanced MRI biomarkers and the methods to combine those with data from psychological assessments, genetics, and histology, as well as the technology readiness level of current data analysis methods, will be assessed by reviewing the state-of-the-art literature. As an example, the ASL-analysis toolbox ExploreASL has recently been launched, allowing the pooling of multi-centre ASL datasets, increasing comparability and narrowing the gap between clinicians, researchers and developers [53]. Special attention will be given to identify existing gaps in the current analysis and use of glioma biomarkers, which is in line with the goals of QIN [46] and OSIPI [49]. Additionally, combined pipelines for advanced MRI in glioma will be distributed on open-access platforms to promote best practices for analysis. Consequently, this working group will lift the current necessity of having in-house expertise and software for the application of advanced MRI biomarkers for glioma diagnostics in both research and clinical settings and work towards analysis standardization.

Working Group ‘Multi-site data integration’ coordinates the development of tools and guidelines for multi-site data integration which will enable the creation of large datasets in glioma diagnostics. This working group will tackle issues on data privacy, data infrastructure and data portability. In the field of data privacy, this working group focuses on standardizing informed consent for patients and participants in glioma imaging research.. Existing standards on data privacy and resources for data exchange will be reviewed, resulting in guidelines and multilingual consent compliant to the General Data Protection Regulation (GDPR). In addition, existing tools and databases for data sharing will be reviewed and guidelines on the use of those data infrastructures will be drafted. Also, data portability issues will be investigated, as pooling existing datasets from multiple imaging sites is complex. For example, the European Network for Brain Imaging of Tumours (ENBIT) offers a repository for brain imaging data, as well as tools for data processing [61, 62]. Such existing datasets are highly heterogeneous, caused by factors such as varying imaging instruments, machine-specific artefacts and differences in MRI-sequences as well as from subtle variations in how the procedures are performed within the clinical setting. Therefore, a common data structure for storage and conveyance of advanced MRI (meta)data will be specified, and the international BIDS standard will be extended for advanced MRI sequences [45]. Other projects facilitating data sharing, such as the development of sequence-specific lexicons, following the example of OSIPI for ASL and DSC/DCE [49], will be supported. These tools will facilitate a multi-site data integration approach to go beyond the use of small local datasets on glioma imaging.

The Working Group ‘Clinical translation’ fosters cross-border information exchange of past, current, and future clinical glioma trials and studies. This group will create a European-wide overview of past and ongoing glioma studies that apply advanced MRI. Next, it will stimulate and coordinate the submission of multi-site grant applications to national, European, and international funding bodies for retrospective and prospective studies, using tools and knowledge provided by the first two Working Groups. This approach will accelerate ground-breaking progress in the development of MRI biomarkers for glioma, as well as the application of radiomics. Additionally, advanced and patient-friendly MRI-protocols and guidelines will be developed, to gather prospective glioma imaging data. Although guidelines were already formulated for conventional MRI in brain tumour imaging [50] as well as for specific advanced MRI techniques in different neurological indications [52], similar recommendations for advanced MRI in glioma are lacking. These protocols and guidelines should both be feasible within an academic and a clinical setting. For this purpose, the Working Group will also seek input from hospital staff members for daily clinical practice, such as nurses and radiographers, as well as from patient organisations, representing the interest of patients diagnosed with glioma.

The fourth Working Group ‘Stakeholder relations’ ensures representation of all relevant stakeholders within GliMR, initiates collaborations with stakeholders inside and outside the network, and coordinates the communication between all stakeholders. Liaisons with stakeholders within the fields of glioma and/or MR imaging will be set-up, including key international consortia and organisations with a focus on glioma and/or imaging biomarkers research, industrial collaborators, clinical practice, local and national European policy makers, patient organisations, as well as relevant ongoing COST Actions groups. Also, other organisations and institutes with complementary expertise and data, such as those related to neuropsychology, neuro-oncology, neurosurgery, molecular biology, pathology, and genetics will be identified and collaborations will be initiated to provide input to the first three Working Groups.

Working Group ‘Dissemination’ supports the other Working Groups in the dissemination of their goals and results, in manners tailored to the research community in- and outside this Action, clinical practice, patient organisations and the general public. This working group maintains several communication channels such as the GliMR website (www.glimr.eu), social media accounts [63] and newsletters, and translates important news into layman’s terms for dissemination via patient organisations and popular media. It will support the other Working Groups in drafting guidelines and best practice documentation to inform participants.

In order to reach the goals of the network and the working groups, GliMR provides opportunities to network, collaborate, discuss, investigate, teach and learn. The Action facilitates and organises (teleconference) meetings and scientific and clinical training schools. Additionally, GliMR has open funding calls for laboratory exchanges (short-term scientific missions) and conference grants for participants affiliated to institutions in inclusiveness target countries, as defined by the COST Association^2^ [64].

## GliMR’s Output After Grant Period One

GliMR’s first grant period ended in April 2020. Throughout this period of seven months, several meetings were held, giving the participants the opportunity to network, collaborate, and discuss glioma research. A kick-off management meeting was organised in September 2019. In October 2019, GliMR hosted a meet-and-greet session during the 36th Annual Scientific Meeting of the European Society for Magnetic Resonance in Medicine and Biology (ESMRMB) (Rotterdam, the Netherlands). Also, a two-day scientific meeting was held in Malta in December 2019, bringing together over 60 participants. Additionally, a call for Short-Term Scientific Missions (STMS) was launched, which offers (early-career) researchers the opportunity to travel to another participating research centre for a certain period, during which a collaboration is set up and a research project is carried out. In total, four STSMs finished successfully despite the COVID-19 pandemic, focussing on the improvement of a DSC analysis pipeline, the prediction of survival using DSC and ASL in glioblastoma, and on the application of machine learning and deep learning on the analysis of perfusion and diffusion MRI as a biomarker for glioma.

Several projects were also initiated by the different Working Groups. The Working Groups ‘Advanced MRI biomarkers for glioma characterisation’ and ‘Clinical translation’ have initiated the development of three literature reviews. These reviews will provide an overview of the currently available advanced MRI techniques for glioma characterisation, MR biomarkers for assessing treatment follow-up, and the evaluation of adverse effects of treatment on healthy brain tissue. Working Group ‘Multi-site data integration’ organised a workshop to develop a GDPR-compliant template of an informed consent form, in collaboration with Open Brain Consent. This work resulted in the creation of a GDPR-compliant consent form and a data transfer agreement, which have been translated into twelve languages,^3^ with more translations planned to be released in the future [65–67] Working Group ‘Stakeholder relations’ has initiated relations with the EIBALL and GLASS, as well as the EORTC Imaging Group. Through its relationship with EIBALL and EORTC, GliMR supported a Horizon 2020 grant application for setting up a European cancer imaging repository. Finally, the Working Group ‘Dissemination’ has set up several communication and dissemination channels, such as the official GliMR website (www.glimr.eu) [63], Twitter (@COST18206) and Instagram page (@glimr2.0), and a YouTube channel [68]. This Working Group is also in charge of creating and releasing a bi-monthly newsletter, issuing press releases and statements (such as the one for the ‘World Cancer Day 2020’), and summarizing the main outputs and news of GliMR.

## Conclusion

Advancing MR imaging is crucial for the diagnosis, prognosis, treatment planning and treatment follow-up of glioma patients and will allow a more personalized disease management approach. However, innovations are hampered by the scattered research landscape of advanced MRI for glioma and the underrepresentation of specialists in this field within current European and international collaborations and organisations. Therefore, GliMR is building a pan-European and multidisciplinary network, to review and share current knowledge, draft and propose consensus guidelines, develop and share tools, and facilitate the execution of multi-centre advanced MRI studies for glioma. All researchers, health care professionals, public and private institutions, patient organisations and policy makers from countries already participating (as illustrated in Fig. 3), as well as from other COST, or even non-COST countries, are welcome to join our Action and Action activities, during the whole duration of the Action. More information on the procedure can be found on the official COST website [69], or by contacting the Action leadership through the website www.glimr.eu.

## Footnotes

1 Austria, Belgium, Bosnia and Herzegovina, Bulgaria, Canada, Croatia, Cyprus, Czech Republic, Denmark, Estonia, France, Germany, Greece, Ireland, Italy, North Macedonia, Malta, Morocco, Norway, Poland, Portugal, Romania, Serbia, Slovakia, Spain, Switzerland, The Netherlands, Turkey, UK, USA.

2 Albania, Bosnia and Herzegovina, Bulgaria, Cyprus, Czech Republic, Estonia, Croatia, Hungary, Lithuania, Latvia, Luxembourg, Malta, Moldova, Montenegro, Poland, Portugal, Romania, Slovenia, Slovakia, Republic of North Macedonia, Republic of Serbia and Turkey.

3 English, Bosnian, Czech, German, Greek, Spanish, Finnish, French, Italian, Dutch, Norwegian, Turkish.
